# Consumer preferences for food allergen labeling

**DOI:** 10.1186/s13223-017-0189-6

**Published:** 2017-04-04

**Authors:** Carlo A. Marra, Stephanie Harvard, Maja Grubisic, Jessica Galo, Ann Clarke, Susan Elliott, Larry D. Lynd

**Affiliations:** 1 School of Pharmacy, Otago University, Dunedin, New Zealand; 2 School of Population and Public Health, Faculty of Medicine, University of British Columbia, Vancouver, BC Canada; 3 Centre for Health Evaluation and Outcome Sciences, Providence Health Care Research Institute, Vancouver, BC Canada; 4 Collaboration for Outcomes Research and Evaluation, Faculty of Pharmaceutical Sciences, University of British Columbia, 2405 Wesbrook Mall, Vancouver, BC V6T 1Z3 Canada; 5 BC Centre for Improved Cardiovascular Health, Vancouver, BC Canada; 6 Population Data BC, Vancouver, BC Canada; 7 Cumming School of Medicine, University of Calgary, Calgary, AB Canada; 8 School of Public Health and Health Systems, University of Waterloo, Waterloo, ON Canada

**Keywords:** Immune system diseases, Hypersensitivity, Hypersensitivity, immediate, Food hypersensitivity, Public health, Health planning, Health services research, Social control, Formal policy, Humans

## Abstract

**Background:**

Food allergen labeling is an important tool to reduce risk of exposure and prevent anaphylaxis for individuals with food allergies. Health Canada released a Canadian food allergen labeling regulation (2008) and subsequent update (2012) suggesting that research is needed to guide further iterations of the regulation to improve food allergen labeling and reduce risk of exposure.

**Objective:**

The primary objective of this study was to examine consumer preferences in food labeling for allergy avoidance and anaphylaxis prevention. A secondary objective was to identify whether different subgroups within the consumer population emerged.

**Methods:**

A discrete choice experiment using a fractional factorial design divided into ten different versions with 18 choice-sets per version was developed to examine consumer preferences for different attributes of food labeling.

**Results:**

Three distinct subgroups of Canadian consumers with different allergen considerations and food allergen labeling needs were identified. Overall, preferences for standardized precautionary and safety symbols at little or no increased cost emerged.

**Conclusion:**

While three distinct groups with different preferences were identified, in general the results revealed that the current Canadian food allergen labeling regulation can be improved by enforcing the use of standardized precautionary and safety symbols and educating the public on the use of these symbols.

## Background

Food allergy (FA) is a public health issue gaining worldwide attention [1–9]. While the overall prevalence of food allergy has been estimated to be approximately 7%, only 3–4% of adults and 5–6% of children have received a physician-confirmed food allergy diagnosis [10–12]. A meta-analysis published in 2007 suggested that the prevalence of food allergy ranges from 3 to 35%, and one Australian study suggested that more than 10% of 1 year olds had a challenge-proven egg allergy [1, 13]. This wide range is a reflection of the varying definition of food allergy (i.e. self-report versus a requirement for symptoms compatible with an IgE-mediated reaction and confirmatory testing), non-reporting of incidents, and respondent bias [1, 14]. Studies suggest that food allergens are the primary cause of anaphylaxis [15–19]. There are about 7% of Canadians with food allergies—among all Canadian children, 1.7% specifically have peanut allergies [14], of which 90% have experienced a severe reaction [14]. A number of US studies have suggested that the incidence of anaphylaxis is increasing and is perhaps as high as 49.8 per 100,000 person-years [15, 20–24]. Additionally, within the last decade, US hospitalizations secondary to food-induced anaphylaxis increased by 350% [25]. Similarly, a study by Ben-Shoshan et al. has revealed that in at least one emergency department in Quebec, Canada, the rate of emergency department visits for anaphylaxis doubled over a 4 year period [26].

There is no cure for food allergy and, thus, strict avoidance of allergenic foods is paramount in symptom prevention [7]. Food allergen labeling is an internationally recognized risk management tool and regulatory policies are being developed to lower food allergen exposure risk for individuals with food allergies [7]. In 2008, the Canadian Minister of Health announced new labeling requirements for food allergens and intolerances contained in pre-packaged foods. A regulatory update was released in August 2012 noting the requirement to list all food allergens, gluten sources, and sulphites in the ingredient lists or in a precautionary statement [27].

Despite the increasing public health concern surrounding food allergies and the recent update in Canadian food allergen labeling regulations, there is scarce information regarding the best way to present allergen information to consumers [28]. Knowledge about consumers’ use of allergen labels may inform regulatory agencies about the appropriate packaging of foods and design of food labels to reduce risk of exposure to food allergens [29–33]. The objective of this study was to use a stated choice experiment to evaluate Canadians’ preferences for different types of food allergen-related information on food labels, and to determine if there are differences in preferences across different types of respondents.

## Methods

### Recruitment and study sample

In order to recruit a representative sample of the Canadian population, respondents were recruited by IPSOS Reid Canada (Vancouver, British Columbia, Canada). Participants were selected from a balanced sample in terms of sociodemographic variables including gender, age, income, level of education, and region within Canada. Subjects were derived from the IPSOS I-Say panel of approximately 300,000 Canadian residents who have agreed to participate in surveys. Respondents were eligible to participate in the study if they were 19 years or older, currently residing in Canada, and were fluent in reading and writing in English.

Ethics approval was obtained from the University of British Columbia Behavioural Research Ethics Board (UBC BREB). Participants were required to provide informed consent prior to study enrolment and were remunerated using IPSOS Reid’s points based system.

### Discrete choice experiment (DCE) questionnaire design

The DCE, one of the most frequently employed techniques to assess consumer preferences, is based on economic theory of choice behaviour and can take into account inter-linked human behaviours [34–37]. In a DCE survey, participants are presented with an array of choice sets representing hypothetical but realistic choice scenarios. Each choice set is composed of different attributes defined by levels that are necessary for decision-making. Participants are asked to make trade-off choices among different combinations of attributes thereby revealing their preferences. By understanding participant preferences between different levels of an attribute, the relative importance of a product characteristic (in this study, the food label), can be determined [38–40].

A qualitative study using focus groups to identify specific attributes of allergen-related food labeling that are most important to consumers was conducted prior to the development of the DCE questionnaire with the approval from the UBC BREB [41]. Eight focus groups were conducted with 2 sample groups of consumers: (1) families with allergic member(s) (n = 26); and (2) the general public (n = 24). Recruitment for the first group was completed through Food Allergy Canada (formerly Anaphylaxis Canada), the largest anaphylaxis support group in Canada. IPSOS Reid conducted recruitment for the second group. The focus groups covered topics related to perceptions of current allergen labeling, information needs, and preferences for allergen labels. Interviews were digitally recorded, transcribed, and analyzed. Based on the results of the qualitative study, four attributes with their respective levels were included in the DCE questionnaire (Table 1).

**Table 1:** Attributes and levels included in the DCE

Attribute	Levels
Precautionary statement	Not suitable for consumers with allergies to peanuts or tree nuts May be present: peanuts and tree nuts May contain traces of peanuts and tree nuts Contains wheat, dairy, peanuts, and tree nuts
Safety statement	Does not contain soy, eggs, fish or shellfish Safety statement not included
Use of symbols	Precautionary symbol Safety symbol Both precautionary and safety symbol No symbols used
Placement of information	Package front Next to ingredients Package front and next to ingredients

**Fig. 1:**
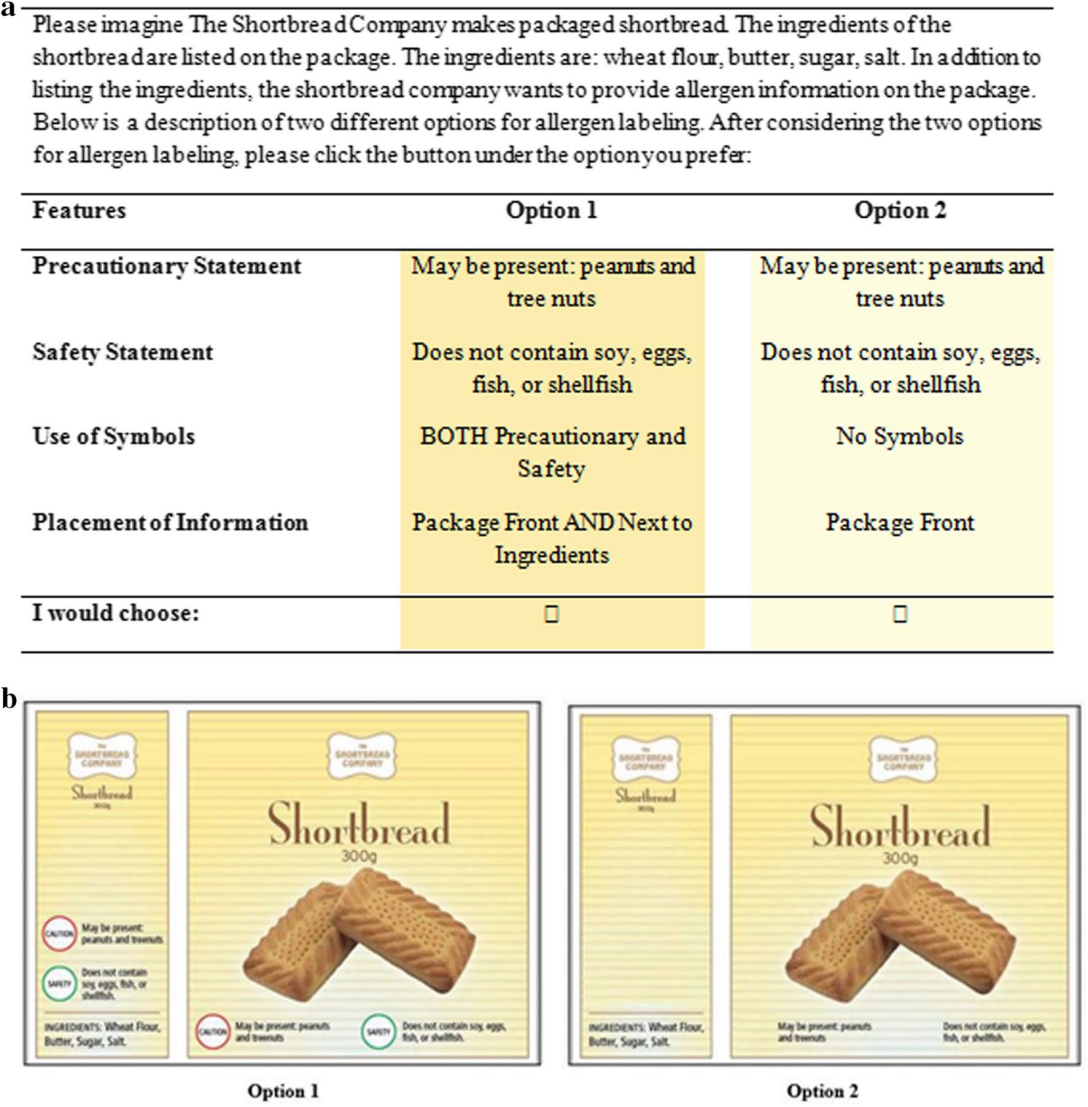
**a** Example of discrete choice experiment choice-set for respondents’ preferences and **b** graphical representation of the label representing attributes in the choice set

Overall, there were 180 possible choice-set combinations, based on 13 levels across four attributes. To reduce the number of choice-sets that each respondent had to complete, a fractional factorial design divided into ten different versions with 18 choice-sets per version was developed. The DCE included 18 choice-sets per respondent in which each respondent was asked to choose between two hypothetical alternatives (Fig. 1a). Furthermore, a picture of the label was presented with each choice set that represented the exact attributes described in the choice set (see Fig. 1b for example). The internal consistency of individuals’ responses was evaluated by including two fixed-repeated choice-sets (not included in the final analysis) in each 18 choice-set version. Prior to recruitment, the DCE was pilot tested in 100 respondents to evaluate the clarity of the questionnaire and that the attribute levels were consistent with the range of preferences. The final survey was designed to take respondents between 15 and 30 min to complete. The final version of the design was checked for orthogonality, level balance, and minimal overlap. In addition to the DCE, the following data was also collected: demographic data (age, gender, province, marital status, household income, level of education, number of children); allergen related data (number of allergen affected individuals in the household, consideration of allergens when buying packaged foods, reasons for considering allergens when buying packaged foods, allergens that a household must avoid, food-related anaphylactic experience by an individual or anyone in their household, and willingness to pay (highest amount an individual was willing to pay above a $500 monthly grocery bill for the inclusion of the allergen information on all food packages, reasons for not wanting to pay any amount for the inclusion of the allergen information on food packages, and the amount an individual was willing to pay above an individual’s annual income taxes in order to include allergen labeling on food packages).

### Statistical analysis

Participants’ baseline characteristics were described using means with standard deviations for continuous variables and frequencies and percentages for categorical variables. Only those respondents who completed all 18 choice sets of the DCE were included in the final analyses. The demographics of consistent and inconsistent respondents (based on the two fixed-repeated choice-sets) were compared to determine if there were any statistically significant differences between these two groups. For the comparison of these two groups, two-sample t tests and χ^2^ tests were used, with the significance level set at 5% (two-tailed). To account for preference heterogeneity between respondents, the respondents’ relative preferences for each level of each attribute were estimated using a latent class model (LCM). Socio-demographic, allergen, and cost variables were investigated for inclusion in the final model based on their influence on class membership. Multiple models with 1–6 latent classes and with and without covariates, all with the same specification other than the number of classes, was evaluated. All attributes and covariates were effect-coded. To determine the most appropriate covariates to include in the final model, a forward selection method with a priori significance set at 5% was used. Selection of the best model (including the number of latent classes) was made based on the Bayesian information criteria (BIC), Akaike information criteria (AIC), and the log-likelihood function. To facilitate explaining the differences in preferences between classes, the relative importance of the attributes across latent classes was examined. The relative importance represents the maximum effects, re-scaled to sum to 1 across attributes within a latent class. All statistics were performed using SAS 9.2 (SAS Institute, Inc., Cary, NC, USA; http://www.sas.com) and Latent GOLD^®^ version 4.5 (Statistical Innovations, Inc., Belmont, MA, USA).

## Results

Of the 1426 respondents who started the online questionnaire, 1100 (77%) completed all 18 choice-sets including the two fixed-repeated choice-sets. Of the 1100 respondents deemed eligible for the study, 108 (10%) did not answer the fixed questions consistently and were classified as inconsistent respondents. The average time to complete the survey was 20 min (SD = 22.6). Some respondents appeared to take a break from the survey, with 67 respondents taking over 24 h to complete the survey. The mean age of the respondents was 46 years (SD = 16), 617 (56%) were female, 173 (16%) had at least a university degree, and 756 (69%) reported having an annual household income of $40,000 or more (Table 2). In addition, 429 (39%) reported at least one allergic individual in their household, 437 (40%) consider allergens when buying food, and 132 (12%) respondents, or someone in their household, had experienced an anaphylactic reaction to a food (Table 3). Correspondingly, 820 (75%) respondents reported being willing to pay for the inclusion of food-allergen information on all food packages (Table 4). There were statistically significant differences between the inconsistent and consistent groups of respondents based on gender, number of allergens per household, number of reasons why respondents consider allergens when buying packaged foods, and all the willingness to pay questions with the exception of not willing to pay more on the respondents’ annual income taxes in order to include allergen labeling on food packages. Therefore, all respondents were included in the final analysis, including a covariate for consistency of response to account for these differences. The only covariate found to be significant was group membership (consistent or inconsistent) thus, the inconsistent group of respondents was excluded from the final model.

**Table 2:** Baseline characteristics of participants

Characteristic	All (N = 1100) N^a^ (%) or mean (SD)	Consistent (N = 992) N^a^ (%) or mean (SD)	Inconsistent (N = 108) N^a^ (%) or Mean (SD)	p value
Age	46.4 (15.6)	46.7 (15.7)	43.8 (14.0)	0.06
Females	617 (56)	567 (57)	50 (46)	0.03
Province				0.97
British Columbia, Alberta, Saskatchewan, Manitoba	403 (37)	364 (37)	39 (36)	
Ontario	508 (46)	457 (46)	51 (47)	
Quebec, Atlantic Provinces, Yukon, Northwest Territories, Nunavut	189 (17)	171 (17)	18 (17)	
Education				0.21
Did not complete high school	81 (7)	74 (7)	7 (6)	
Completed high school and/or some college or trade	846 (77)	756 (76)	90 (83)	
Completed university or professional degree	173 (16)	162 (16)	11 (10)	
Income				0.30
<$4000	339 (31)	305 (31)	34 (31)	
$40,000–$80,000	405 (37)	359 (36)	46 (43)	
>$80,000	351 (32)	323 (33)	28 (26)	
Children (yes)	334 (31)	297 (30)	37 (34)	0.37
Marital status				0.33
Single, widowed, divorced, separated	359 (33)	328 (33)	31 (29)	
Married, domestic partnership	734 (67)	657 (67)	77 (71)	
Household size				0.40
One person	166 (15)	147 (15)	19 (18)	
Family of two	365 (33)	325 (33)	40 (37)	
Three or more family members	564 (52)	515 (52)	49 (45)	

*SD* standard deviation

^a^Not all respondents responded to all questions, therefore, the N varies

**Table 3:** Participant experience with food allergens

Variables	All (N = 1100) N (%)	Consistent (N = 992) N (%)	Inconsistent (N = 108) N (%)	p value
Have you or has anyone in your household experienced an anaphylactic reaction to a food (yes)	132 (12)	123 (12)	9 (8)	0.22
Do you consider allergens when buying food (yes)	437 (40)	402 (41)	35 (32)	0.10
Number of allergens per household				0.004
No allergens	671 (61)	596 (60)	75 (69)	
One allergen	230 (21)	204 (21)	26 (24)	
Two or more allergens	199 (18)	192 (19)	7 (6)	
Allergen(s) households if any must avoid				
N/A	671 (61)	596 (60)	75 (69)	
Peanuts	171 (16)	157 (16)	14 (13)	
Tree nuts	90 (8)	85 (9)	5 (5)	
Milk	99 (9)	95 (10)	4 (4)	
Egg	45 (4)	44 (4)	1 (1)	
Fish	35 (3)	34 (3)	1 (1)	
Shellfish	95 (9)	94 (9)	1 (1)	
Soy	16 (1)	15 (2)	1 (1)	
Wheat	67 (6)	61 (6)	6 (6)	
Sesame seeds	20 (2)	19 (2)	1 (1)	
Other	95 (9)	87 (9)	8 (7)	
Number of reasons why respondents consider allergens when buying packaged foods				0.02
Do not consider allergens	608 (55)	535 (54)	73 (68)	
One reason	347 (32)	321 (32)	26 (24)	
Two or more reasons	145 (13)	136 (14)	9 (8)	
Reasons why respondents consider allergens when buying packaged foods				
N/A	608 (55)	535 (54)	73 (68)	
I have a food allergy	156 (14)	143 (14)	13 (12)	
One or more of my children has a food allergy	82 (7)	75 (8)	7 (6)	
My spouse or partner has a food allergy	72 (7)	63 (6)	9 (8)	
Another member of my household has a food allergy	55 (5)	53 (5)	2 (2)	
A friend who visits my home has a food allergy	112 (10)	105 (11)	7 (6)	
My child’s school has allergen restrictions	136 (12)	129 (13)	7 (6)	
My workplace has allergen restrictions	44 (4)	43 (4)	1 (1)	
Other	47 (4)	46 (5)	1 (1)

**Table 4:** Summary of the cost questions

Cost questions	All (N = 1100) N (%)	Consistent (N = 992) N (%)	Inconsistent (N = 108) N (%)	p value
Above an average of $500 per month spent on food, what is the most you would be willing to pay every month for the inclusion of the allergen information on all food packages?				0.0109
$0	280 (25)	247 (25)	33 (31)	
Between $0 and $10	424 (39)	393 (40)	31 (28)	
Between $10 and $50	236 (21)	217 (22)	19 (17)	
More than $50	160 (15)	135 (14)	25 (23)	
You specified that you are willing to pay $0 for the inclusion of the allergen information on food packages—what is the reason?				0.017
The food allergen labeling is of no value to me or my family	88 (31)	71 (29)	17 (50)	
I cannot afford to pay more	63 (23)	54 (22)	9 (26)	
The government or another group in society should pay for it	57 (20)	54 (22)	3 (9)	
Other	72 (26)	68 (28)	4 (12)	
Are you willing to pay more on your annual income taxes in order to include allergen labeling on food packages?				
No	871 (79)	787 (79)	84 (78)	0.71
Yes	229 (21)	205 (21)	24 (22)	
$0	11 (5)	8 (3)	3 (13)	0.03
Between $0 and $10	67 (29)	56 (27)	11 (46)	
Between $10 and $50	84 (37)	80 (39)	4 (17)	
More than $50	67 (29)	61 (30)	6 (25)	

A LCM including explanatory variables compared to a model including only the attribute responses improved the model fit. After considering the goodness of fit statistics, the interpretability and relative sizes of the classes, a 3-class model was selected as best representing respondents’ preferences (Table 5). Most parameter values for the choice model were significant at the 5% level. Due to missing data on age, gender and marital status, only 985 respondents were included in the final latent class analysis.

**Table 5:** Preferences for each level of each attribute based on the latent class analysis

Attribute	Class 1 Mean (SE)	Class 2 Mean (SE)	Class 3 Mean (SE)
Class probabilities	0.44 (0.0246)	0.38 (0.0241)	0.18 (0.0152)
Precautionary statement			
Not suitable for consumers with allergies to peanuts or tree nuts	0.162^†^ (0.0453)	0.252^†^ (0.0964)	−0.0078 (0.0612)
May be present: peanuts and tree nuts	−0.116^†^ (0.0396)	−0.109* (0.0736)	0.294^†^ (0.0614)
May contain traces of peanuts and tree nuts	0.040 (0.0398)	−0.0042 (0.0753)	−0.032 (0.0572)
Manufactured in a facility that also processes peanuts and tree nuts	0.119^†^ (0.0447)	−0.0057 (0.0799)	−0.136^†^ (0.0659)
Contains wheat, dairy, peanuts and tree nuts	−0.205^†^ (0.0419)	−0.133* (0.0843)	−0.119^†^ (0.0572)
Safety statement			
Does not contain soy, eggs, fish, or shellfish	0.220^†^ (0.0394)	1.073^†^ (0.0653)	−0.318^†^ (0.0497)
Not included	−0.220^†^ (0.0394)	−1.073^†^ (0.0653)	0.318^†^ (0.0497)
Use of symbols			
Precautionary symbol	0.433^†^ (0.0502)	0.094 (0.0959)	0.268^†^ (0.0625)
Safety symbol	−0.305^†^ (0.0544)	−0.084 (0.1032)	−0.071 (0.0749)
Both precautionary and safety symbol	0.796^†^ (0.0608)	1.069^†^ (0.1316)	−0.285^†^ (0.0823)
No symbols used	−0.925^†^ (0.0594)	−1.078^†^ (0.0861)	0.088* (0.0691)
Placement of information			
Package front	−0.054^‡^ (0.0299)	−0.227^†^ (0.0486)	−0.269^†^ (0.0413)
Next to ingredients	−0.296^†^ (0.0352)	−0.234^†^ (0.0602)	0.355^†^ (0.0421)
Package front and next to ingredients	0.350^†^ (0.0318)	0.461^†^ (0.0621)	−0.086^†^ (0.0406)
Covariate parameter estimates			
Intercept	0.502^†^ (0.1968)	−0.780^†^ (0.2105)	0.278* (0.2254)
Age	−0.0025 (0.0037)	0.0225^†^ (0.0038)	−0.020^†^ (0.0045)
Marital status			
Single, widowed, divorced, separated	−0.058 (0.0579)	0.130^†^ (0.0599)	−0.072* (0.0679)
Married, domestic partnership	0.058 (0.0579)	−0.130^†^ (0.0599)	0.072* (0.0679)
Education			
Did not complete high school	−0.091 (0.1482)	0.309^†^ (0.1390)	−0.218* (0.1759)
Completed high school and/or some college or trade	0.058 (0.0901)	0.112* (0.0894)	−0.169* (0.1054)
Completed university or professional degree	0.033 (0.1120)	−0.420^†^ (0.1199)	0.388^†^ (0.1248)
Province			
BC, AB, SA, MA	−0.064 (0.0758)	−0.138^‡^ (0.0793)	0.202^†^ (0.0869)
Ontario	0.083* (0.0734)	−0.017 (0.0759)	−0.066 (0.0879)
Quebec, Atlantic Provinces, Yukon, Northwest Territories, Nunavut	−0.019 (0.0937)	0.155* (0.0966)	−0.136* (0.1117)
Number of reasons to consider allergens			
No reasons	−0.158* (0.0993)	0.091 (0.1071)	0.067 (0.1191)
One reason	0.232^†^ (0.0959)	−0.216^†^ (0.1059)	−0.016 (0.1206)
Two or more reasons	−0.074 (0.1026)	0.125* (0.1019)	−0.051 (0.1295)
Do you consider allergens			
Yes	−0.066 (0.0745)	0.233^†^ (0.0790)	−0.167^†^ (0.0893)
No	0.066 (0.0745)	−0.233^†^ (0.0790)	0.167^†^ (0.0893)
Cost above $500 for groceries			
$0	−0.067 (0.0932)	−0.243^†^ (0.0984)	0.309^†^ (0.1033)
Less than $10	0.027 (0.0824)	−0.064 (0.0848)	0.037 (0.0985)
Between $10 and $50	0.062 (0.1033)	0.223^†^ (0.1030)	−0.285^†^ (0.1315)
More than $50	−0.023 (0.1201)	0.084 (0.1184)	−0.061 (0.1449)
Log-likelihood	−7538.16		
No. of individuals	985		
No. of observations	15,760		

*SE* standard error

^†^p value <0.05; ^‡^ p value <0.10; * p value <0.15

**Fig. 2:**
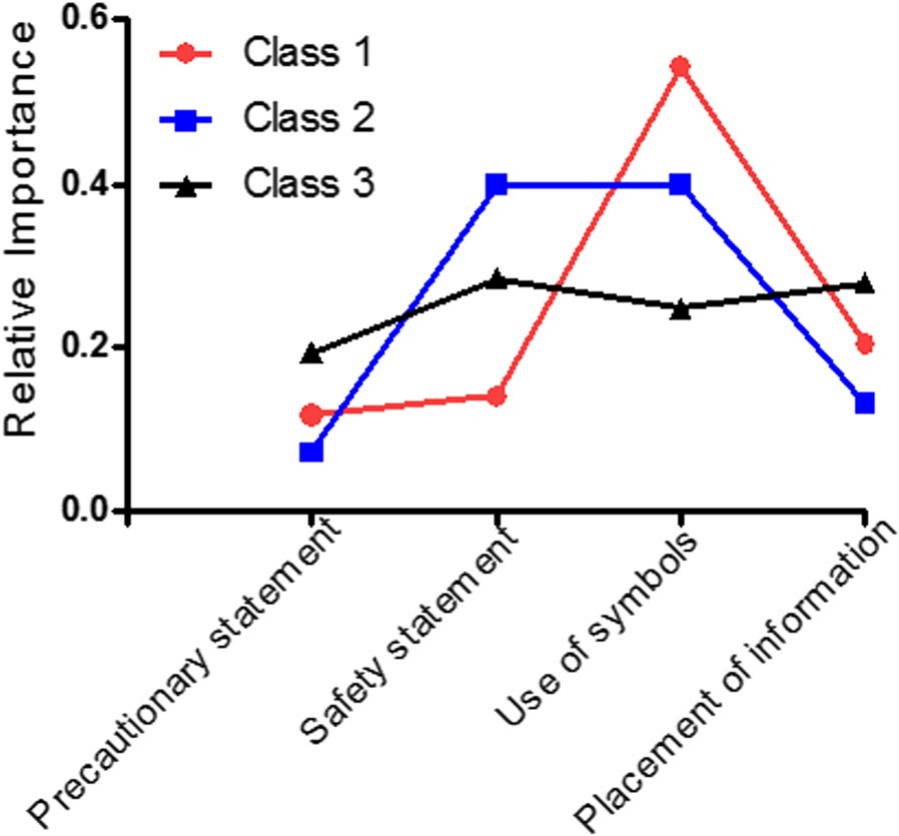
Relative importance of attributes by class. The preference weights of maximum effect of each attribute are rescaled such that they sum to one across all attributes for each latent class

The relative preferences and a weighted average over all classes were calculated for each attribute. Use of symbols was the most important attribute accounting for 43.5% of the variance explained, on average. The presence of a safety statement accounted for 26.4%. Placement of information and the precautionary statement accounted for 18.9 and 11.3%, respectively. The results indicate that class 1 respondents (with the probability of being in class 1 being the greatest, 44%) had the strongest negative preference for no symbols used (−0.925), and their strongest positive preference was for both a precautionary and safety symbol (0.796). Thus, the presence of symbols was the most important attribute for those in class 1 (Fig. 2). Class 2 respondents (with the probability of being in class 2, 38%) had even stronger preferences for having both precautionary and safety symbols on the label (1.069), but the presence of the safety statement, “Does not contain soy, eggs, fish, or shellfish” was equally preferred (1.073). Finally, the most important attribute level for class 3 respondents (with the probability of being in class 3, 18%) was the placement of the allergen information next to ingredients on a food package (0.355), but overall, the preferences of class 3 respondents did not differ across the attributes (Fig. 2). Of note, those respondents who consider allergens when making food purchases preferred the ‘precautionary’ statements the least.

Table 5 also shows that the inclusion of the covariates in the model significantly improved the model fit of the three latent class segments compared to not including any covariates. A positive and significant coefficient of a covariate indicates a greater tendency for respondents in that class to have a particular characteristic. Seven covariates were found to be significant at 0.05 significance level: age, marital status, education, province, consideration of allergens, number of reasons to consider allergens, and amount willing to spend for the inclusion of allergen information. The respondents who identified the use of symbols as most important (class 1 respondents) were most likely to consider allergens for only one reason; no other covariates were significant. Class 2 respondents, who were most concerned about both the presence of a safety statement and the use of symbols, were also most likely to consider allergens when shopping for food and were willing to pay an additional $10–$50 above the $500 monthly grocery expense on food for the inclusion of the allergen information. They were also more likely to be older, single/widowed/divorced or separated, and have not completed high school. Class 3 respondents, who chose placement of information as most important, were the most likely not to consider allergens at all when purchasing food and were not willing to pay any amount above a $500 monthly grocery expense on food for the inclusion of the allergen information. They also tended to be younger, completed at least a university undergraduate degree, and live in one of the Western provinces.

In terms of their willingness to pay, the majority of consistent respondents were willing to pay up to $10 extra per month for groceries for the inclusion of allergen labels on food. Consistent respondents who were not willing to pay an additional cost either could not afford to pay more, thought that allergen labeling was of no value to their household, that other groups should be responsible for the cost, or were not willing to pay more for other unknown reasons. The respondents’ characteristics associated with their cost preferences become apparent in the LCM analysis. Class 2 respondents were willing to pay $10–$50 more for their monthly groceries for the inclusion of allergen labels on food even though their income potential, as evidenced by their education level, may be lower. Conversely, class 3 respondents reported a higher income but were not willing to pay any additional cost for the inclusion of allergen food labels. This group did not have a need for allergen labels. It would seem that an individual’s willingness to pay an additional cost for the inclusion of food allergen labels is not determined by their income potential but rather their allergen labeling needs.

## Discussion

This is the first study in Canada to examine consumer preferences regarding food allergen labeling of pre-packaged foods using a DCE, one of the most effective methodological techniques, consistent with economic theory. The DCE and LCM account for the heterogeneity of food labeling preferences among Canadian respondents thereby reducing the potential for bias and loss of information related to food labeling regulatory practices. Overall, the majority of respondents prefer some type of allergen labeling. The use of symbols was the most important food allergen-labeling attribute for those in class 1 (44%) and the use of symbols and a safety statement were equally important to those in class 2 (38%) of respondents, with respondents in both classes preferring both precautionary and safety symbols. Those in class 3 (18%) were essentially indifferent to allergen labeling.

Overall, the second most important food allergen labeling attribute identified was the use of the safety statement “does not contain soy, eggs, fish or shellfish”. Placement of information and the use of precautionary statements were the third and fourth most important attributes. The use of precautionary statements, such as “may contain traces of peanuts” may be preferred the least due to the ambiguity of the statement and the necessity of consumers to use their discretion in choosing or avoiding these products. Different precautionary expressions may be confusing and the level of allergic risk associated with each expression may be deemed unascertainable [30, 31]. Additionally, these statements may be viewed as causing unnecessary diet restrictions as opposed to providing informed food choices [28, 29, 31, 42].

We are not aware of any other quantitative studies that have specifically evaluated consumers preferences for food allergen labeling. Although this is the first study that looked at consumer food allergen labeling preferences using the DCE, our results are consistent with qualitative studies investigating similar allergen labeling questions [28, 29]. In some qualitative studies, participants were interviewed and observed during the course of grocery shopping. For instance, in an Ontario study, Chow et al. found that parents of children with food allergies trusted products with allergen symbols and found them easily understandable [28]. Similarly, Cornelisse-Vermaat et al. reported that parents preferred labels with both allergen symbols and textual allergen information [29]. Perhaps, similar to nutrition labeling studies, symbol use in allergen labeling is favoured because it is requires less information processing [29, 40, 43, 44].

The results of the DCE suggest that consumers’ preferences for allergen labels on foods varied widely. A 3-class model appeared to best fit our data and the class memberships were associated with seven measurable sociodemographic factors. Class 1 individuals, accounting for 44% of respondents, predominantly reported considering allergens when buying food due to one unspecified reason which presumably was associated with someone in their household having a food allergy. This group preferred the use of both precautionary and safety symbols on food allergen labels. Respondents who preferred the use of safety statements and both precautionary and safety symbols accounted for 38% of respondents and fell in the class 2 group. On average, these individuals were older, not in a partnership, had not completed high school or post-secondary education, and considered allergens for more than one unspecified reasons. It is possible that these individuals live or work in a care facility or work in a service industry where food allergen vigilance is typically high. Class 3, accounting for 18% of respondents, had the highest education levels of the three classes. This class did not consider allergens when buying foods, which could also explain why this group was not willing to pay anything for the inclusion of allergen information and that there was really no difference in the relative importance of each attribute versus classes 1 and 2 who likely had a specific need for food allergen information.

The inclusion of food allergen information could result in increased costs to the food industry which would then presumably be passed on to the consumer, thus it was important to not only evaluate Canadians’ preferences for labeling but also their willingness to pay. Our results showed, as one might expect, that those without a specific need for food allergen labeling (i.e. class 3 respondents) were not willing to pay more for food to have specific allergen labeling on foods. However, the majority of consistent respondents were willing to pay $0–$10 for the inclusion of allergen labels on food and it appears that an individual’s willingness to pay an additional cost for the inclusion of food allergen labels is not determined by their income but rather their allergen needs.

A 2011 Canadian study found that consumers do not trust Canadian food allergen labels rendering them largely ineffective [28]. The updated Canadian food allergen labeling regulation, released in August 2012, addressed the need to standardize the location of allergen information on food labels as well as the need for specificity of allergen sources [27]. Our research suggests that the regulation has a number of outstanding issues to address. First, consumers preferred the use of symbols on labels; however, the current Canadian regulation does not enforce the use of any symbols on allergen labels [27]. These symbols need to be standardized and the public educated about their significance. Second, consumers who consider allergens preferred the use of precautionary statements the least. While we did not specifically explore the reasons for choosing one format over another, previous studies suggest that these statements provide no definite allergen content information apart from cross-contamination thereby limiting food choices of consumers [28, 29, 31, 42, 45]. This limitation causes consumers to take on more risk and rely on product or brand experience as opposed to allergen labels in decision making [28, 29, 45]. Additionally, the terminology within precautionary statements is currently not standardized across manufacturers leaving consumers confused [29, 30, 45].

As with any questionnaire-based research, there are several limitations. Firstly, while the questionnaire was only administered in English, we do not anticipate that this would have biased the results in any way. It is also important to consider that the responses are based on a stated choice experiment and not on actual choices. However, the results provide a valid evaluation of relative preferences for each labeling attribute, which may direct allergen-labeling regulations towards a standardized and accepted food allergen label. Additionally, respondents were recruited through an IPSOS panel and only included respondents who had computer access. While this could result in a selection bias of respondents, we feel that these preferences do reflect the preferences of the average Canadian household.

While it is never possible to know if respondents completely understood the task or questions, the results do provide an assessment based on their face validity, e.g. those with a need for allergen avoidance had stronger preferences. Furthermore, we incorporated two fixed repeated choice questions in the final version of the survey which showed that approximately 10% of respondents were considered inconsistent and were deemed to not have made meaningful choices. Data from these respondents were therefore excluded from analysis, contributing to the validity of the final results. Finally, our results are also in agreement with earlier qualitative findings, which supported the theoretical validity of our DCE methodology.

Labeling is the most important risk management tool in reducing exposure to allergens. Studies have shown that the current labeling system is insufficient in preventing allergen exposure [28, 29, 41]. Labels that are ambiguous and confusing have led to decreased consumer confidence in allergen labeling and increased risk exposure. Our results suggest that labels need to be standardized and intuitive to make them easily understandable by the broader public. A more definite allergen content statement is preferred as well as the use of symbols to communicate allergen information. Further studies are required to determine reasons behind the consumers’ stated preferences and to compare these stated preferences with actual decisions. The current iteration of the Canadian regulation addressed the need for allergen content specificity on the labels however the results of this study identify additional changes that will make Canadian allergen food labels more effective according to stated consumer preferences.

## Key messages

- Canadian consumers’ food allergen vigilance, labeling preferences, and willingness to spend on improved labeling were investigated.
- Three distinct classes of consumers emerged with different need and preferences for food labeling.
- Canadian consumers identified preferences for (1) standardized precautionary and safety statements and symbols; (2) the use of symbols more than statements; (3) little or no increase in cost for improved food allergen labeling.
- While the majority of respondents had strong preferences for safety statements and use of symbols, a small proportion of respondents appeared to be indifferent to food allergen labelling and were no likely to consider allergens when buying foods.

## Capsule summary

Canadian food allergen labeling regulations can be improved to reduce food allergen exposure risk by standardizing the precautionary and safety labeling and relying more on symbols than statements.

## Abbreviations

FA: food allergy
DCE: discrete choice experiment
BREB: behavioural research ethics board
LCM: latent class model
BIC: Bayesian information criteria
AIC: Akaike information criteria

## References

[1] Rona RJ, Keil T, Summers C, Gislason D, Zuidmeer L, Sodergren E, Sigurdardottir ST, Lindner T, Goldhahn K, Dahlstrom J (2007). The prevalence of food allergy: a meta-analysis. J Allergy Clin Immunol.

[2] Anandan C, Sheikh A (2005). European developments in labelling allergenic foods. BMJ.

[3] Sampson HA (2004). Update on food allergy. J Allergy Clin Immunol.

[4] Grundy J, Matthews S, Bateman B, Dean T, Arshad SH (2002). Rising prevalence of allergy to peanut in children: data from 2 sequential cohorts. J Allergy Clin Immunol.

[5] Sicherer SH, Munoz-Furlong A, Sampson HA (2003). Prevalence of peanut and tree nut allergy in the United States determined by means of a random digit dial telephone survey: a 5-year follow-up study. J Allergy Clin Immunol.

[6] Sicherer SH, Munoz-Furlong A, Burks AW, Sampson HA (1999). Prevalence of peanut and tree nut allergy in the US determined by a random digit dial telephone survey. J Allergy Clin Immunol.

[7] Gendel SM (2013). The regulatory challenge of food allergens. J Agric Food Chem.

[8] O’Hara NN, Roy L, O’Hara LM, Spiegel JM, Lynd LD, FitzGerald JM, Yassi A, Nophale LE, Marra CA (2015). Healthcare worker preferences for active tuberculosis case finding programs in South Africa: a best–worst scaling choice experiment. PLoS ONE.

[9] Guo N, Marra CA, FitzGerald JM, Elwood RK, Anis AH, Marra F (2011). Patient preference for latent tuberculosis infection preventive treatment: a discrete choice experiment. Value Health.

[10] Soller L, Ben-Shoshan M, Harrington DW, Fragapane J, Joseph L, St Pierre Y, Godefroy SB, La Vieille S, Elliott SJ, Clarke AE (2012). Overall prevalence of self-reported food allergy in Canada. J Allergy Clin Immunol.

[11] Venter C, Pereira B, Voigt K, Grundy J, Clayton CB, Higgins B, Arshad SH, Dean T (2008). Prevalence and cumulative incidence of food hypersensitivity in the first 3 years of life. Allergy.

[12] Zuberbier T, Edenharter G, Worm M, Ehlers I, Reimann S, Hantke T, Roehr CC, Bergmann KE, Niggemann B (2004). Prevalence of adverse reactions to food in Germany—a population study. Allergy.

[13] Allen KJ, Koplin JJ (2015). Why does Australia appear to have the highest rates of food allergy?. Pediatr Clin N Am.

[14] Ben-Shoshan M, Harrington DW, Soller L, Fragapane J, Joseph L, St Pierre Y, Godefroy SB, Elliott SJ, Clarke AE (2010). A population-based study on peanut, tree nut, fish, shellfish, and sesame allergy prevalence in Canada. J Allergy Clin Immunol.

[15] Decker WW, Campbell RL, Manivannan V, Luke A, St Sauver JL, Weaver A, Bellolio MF, Bergstralh EJ, Stead LG, Li JT (2008). The etiology and incidence of anaphylaxis in Rochester, Minnesota: a report from the Rochester Epidemiology Project. J Allergy Clin Immunol.

[16] Simon MR, Mulla ZD (2008). A population-based epidemiologic analysis of deaths from anaphylaxis in Florida. Allergy.

[17] Pumphrey R (2004). Anaphylaxis: can we tell who is at risk of a fatal reaction?. Curr Opin Allergy Clin Immunol.

[18] Liew WK, Williamson E, Tang ML (2009). Anaphylaxis fatalities and admissions in Australia. J Allergy Clin Immunol.

[19] Piromrat K, Chinratanapisit S, Trathong S (2008). Anaphylaxis in an emergency department: a 2-year study in a tertiary-care hospital. Asian Pac J Allergy Immunol.

[20] Yocum MW, Khan DA (1994). Assessment of patients who have experienced anaphylaxis: a 3-year survey. Mayo Clin Proc.

[21] Yocum MW, Butterfield JH, Klein JS, Volcheck GW, Schroeder DR, Silverstein MD (1999). Epidemiology of anaphylaxis in Olmsted County: a population-based study. J Allergy Clin Immunol.

[22] Sheikh A, Alves B (2000). Hospital admissions for acute anaphylaxis: time trend study. BMJ.

[23] Lin RY, Anderson AS, Shah SN, Nurruzzaman F (2008). Increasing anaphylaxis hospitalizations in the first 2 decades of life: New York State, 1990–2006. Ann Allergy Asthma Immunol.

[24] Calvani M, Di Lallo D, Polo A, Spinelli A, Zappala D, Zicari AM (2008). Hospitalizations for pediatric anaphylaxis. Int J Immunopathol Pharmacol.

[25] Branum AM, Lukacs SL (2009). Food allergy among children in the United States. Pediatrics.

[26] Hochstadter E, Clarke A, De Schryver S, LaVieille S, Alizadehfar R, Joseph L, Eisman H, Ben-Shoshan M (2016). Increasing visits for anaphylaxis and the benefits of early epinephrine administration: a 4-year study at a pediatric emergency department in Montreal, Canada. J Allergy Clin Immunol.

[27] Canadian Food Inspection Agency. Food allergies and allergen labelling. 2016. Government of Canada website, http://www.inspection.gc.ca/food/labelling/core-requirements/ingredients/allergen-labelling/eng/1332352596437/1332352683099. Accessed 23 Feb 2017.

[28] Chow YLB. Everybody else got to have this cookie: the effects of food allergen labels on the well-being of Canadians (Masters thesis, McMaster University, Hamilton, ON). 2011. http://hdl.handle.net/11375/11078.

[29] Cornelisse-Vermaat JR, Voordouw J, Yiakoumaki V, Theodoridis G, Frewer LJ (2008). Food-allergic consumers’ labelling preferences: a cross-cultural comparison. Eur J Public Health.

[30] Turner PJ, Kemp AS, Campbell DE (2011). Advisory food labels: consumers with allergies need more than “traces” of information. BMJ.

[31] Noimark L, Gardner J, Warner JO (2009). Parents’ attitudes when purchasing products for children with nut allergy: a UK perspective. Pediatr Allergy Immunol.

[32] Sheth SS, Waserman S, Kagan R, Alizadehfar R, Primeau MN, Elliot S, St Pierre Y, Wickett R, Joseph L, Harada L (2010). Role of food labels in accidental exposures in food-allergic individuals in Canada. Ann Allergy Asthma Immunol.

[33] Ben-Shoshan M, Sheth S, Harrington D, Soller L, Fragapane J, Joseph L, St Pierre Y, La Vieille S, Elliott S, Waserman S (2012). Effect of precautionary statements on the purchasing practices of Canadians directly and indirectly affected by food allergies. J Allergy Clin Immunol.

[34] Louviere J, Flynn T (2010). Discrete choice experiments are not conjoint analysis. Choice Model.

[35] Lancsar E, Louviere J, Flynn T (2007). Several methods to investigate relative attribute impact in stated preference experiments. Soc Sci Med.

[36] Lancsar E, Louviere J (2008). Conducting discrete choice experiments to inform healthcare decision making: a user’s guide. Pharmacoeconomics.

[37] Lagarde M, Blaauw D (2009). A review of the application and contribution of discrete choice experiments to inform human resources policy interventions. Hum Resour Health.

[38] Guimaraes C, Marra CA, Gill S, Simpson S, Meneilly G, Queiroz RH, Lynd LD (2011). A discrete choice experiment evaluation of patients’ preferences for different risk, benefit, and delivery attributes of insulin therapy for diabetes management. Patient Prefer Adherence.

[39] Laba TL, Brien JA, Fransen M, Jan S (2013). Patient preferences for adherence to treatment for osteoarthritis: the MEdication Decisions in Osteoarthritis Study (MEDOS). BMC Musculoskelet Disord.

[40] Hoefkens C, Veettil PC, Van Huylenbroeck G, Van Camp J, Verbeke W (2012). What nutrition label to use in a catering environment? A discrete choice experiment. Food Policy.

[41] Brown KM, Fenton NE, Lynd LD, Marra CA, FitzGerald JM, Harvard SS, Rosenthal M, Chow BYL, Clarke AE, Elliott SJ (2015). Canadian policy on food allergen labelling: consumers’ perspectives regarding unment needs. Univers J Public Health.

[42] Gupta RS, Springston EE, Kim JS, Smith B, Pongracic JA, Wang X, Holl J (2010). Food allergy knowledge, attitudes, and beliefs of primary care physicians. Pediatrics.

[43] Campos S, Doxey J, Hammond D (2011). Nutrition labels on pre-packaged foods: a systematic review. Public Health Nutr.

[44] Feunekes GI, Gortemaker IA, Willems AA, Lion R, van den Kommer M (2008). Front-of-pack nutrition labelling: testing effectiveness of different nutrition labelling formats front-of-pack in four European countries. Appetite.

[45] Barnett J, Muncer K, Leftwich J, Shepherd R, Raats MM, Gowland MH, Grimshaw K, Lucas JS (2011). Using ‘may contain’ labelling to inform food choice: a qualitative study of nut allergic consumers. BMC Public Health.

